# *GCN5* Is a Positive Regulator of Origins of DNA Replication in *Saccharomyces cerevisiae*

**DOI:** 10.1371/journal.pone.0008964

**Published:** 2010-01-29

**Authors:** Maria Claudia Espinosa, Muhammad Attiq Rehman, Patricia Chisamore-Robert, Daniel Jeffery, Krassimir Yankulov

**Affiliations:** Department of Molecular and Cellular Biology, University of Guelph, Guelph, Ontario, Canada; University of Minnesota, United States of America

## Abstract

*GCN5* encodes one of the non-essential Histone Acetyl Transferases in *Saccharomyces cerevisiae*. Extensive evidence has indicated that *GCN5* is a key regulator of gene expression and could also be involved in transcriptional elongation, DNA repair and centromere maintenance. Here we show that the deletion of *GCN5* decreases the stability of mini-chromosomes; that the tethering of *Gcn5p* to a crippled origin of replication stimulates its activity; that high dosage of *GCN5* suppresses conditional phenotypes caused by mutant alleles of *bona fide* replication factors, *orc2-1*, *orc5-1* and *mcm5-461*. Furthermore, Gcn5p physically associates with origins of DNA replication, while its deletion leads to localized condensation of chromatin at origins. Finally, *Δgcn5* cells display a deficiency in the assembly of pre-replicative complexes. We propose that *GCN5* acts as a positive regulator of DNA replication by counteracting the inhibitory effect of Histone Deacetylases.

## Introduction

DNA replication in *Saccharomyces cerevisiae* initiates at discrete origins referred to as Autonomously Replicating Sequences (*ARS*). Origins share an essential core element called *ACS* (*ARS* Consensus Sequence), which binds the Origin Recognition Complex (ORC). In early G1 phase Cdc6p and six MCM (MiniChromosome Maintenance) proteins (MCM2-7) join ORC to form pre-replicative complexes [1]. In S phase these complexes are activated by protein kinases to initiate DNA replication [1].

It is granted that histone acetylation can play a key role in the regulation of origins of DNA replication [2], [3]. Convincing evidence has shown that in *S.cerevisiae* two major Histone-DeACetylases (HDAC), *RPD3* and *SIR2*, act as negative regulators of origins [4], [5], [6], [7]. It has been postulated that their inhibitory role is mediated by global deacetylation rather than local recruitment and retention of HDACs to replication foci. However, a recent study suggests that *RPD3* may be targeted to multiple non-telomeric origins but the mechanism of recruitment remains elusive [7]. A link between histone deacetylation by Rpd3p and negative impact on origin activity has also been proposed in *Drosophila* [8]. At the same time, another HDAC, *HST1*, surprisingly acted as a stimulator of certain origins [9] thus suggesting a more complex cross-talk between histone acetylation and replication. Nevertheless, the prevailing notion is that deacetylation of histones has a negative impact on origin activity [3].

It is conceivable that the opposing activity of Histone Acetyl Transferases (HAT) stimulates origins of DNA replication, but details about the HATs involved are sparse. For example, in *S.cerevisiae* the tethering of *Gcn5p* to the late origin *ARS1412* causes it to fire earlier [4]. Similarly, the tethering of Chameau (a HAT) to a chromosomal origin in *Drosophila* stimulates the activity of this origin [8] thus reiterating the general link between histone acetylation and activity of origins. Several recent studies in human cells have demonstrated that the HAT HBO1 is involved in the loading of MCM proteins on chromatin in early G1 phase [10], [11], [12] and in the progression of replication forks in S-phase [13]. Furthermore, HBO1 seems to respond to p53 and FAD24 to halt cell cycle progression [14], [15]. HBO1 has no obvious structural homologue in *S.cerevisiae*, however the Sas2p-containing HAT complex NuA3 has been proposed as a remote functional homologue [16], [17]. Interestingly, at semi-permissive temperature the deletion of *SAS2* exacerbates the low origin activity caused by a mutation in *orc2* and delays progression through S-phase in conjunction with *orc2* mutations [18]. Another recent study has documented an interaction between Hat1p and ORC and has shown preferential association of Hat1p with origins at the time of their activation [19].

In this study we compared the effects of the deletions or mutations in several HATs including *SAS2* and *HAT1* on DNA replication in *S.cerevisiae*. We obtained several lines of evidence indicating that *GCN5* is a major positive regulator of origins of DNA replication.

## Materials and Methods

The yeast strains used in this study are listed in Table 1. Yeast cells were routinely grown in YPD or SC media at 23°C except in Table 2 as indicated.

**Table 1:** Yeast strains used in this study.

Strain	Genotype	Reference
*W303*	*ade2-1 trp1-1 can1-100 leu2-3,112 his3-11,15 ura3-1 MAT* ***a***	
*mcm5-461*	*mcm5-461 ade2-1 trp1-1 can1-100 leu2-3,112 his3-11,15 ura3-1 MAT* ***α*** *, isogenic to W303*	[29]
*orc2-1*	*orc2-1 ade2-1 trp1-1 can1-100 leu2-3,112 his3-11,15 ura3-1 MAT* ***a*** *, isogenic to W303*	[31]
*orc5-1*	*orc5-1 ade2-1 trp1-1 can1-100 leu2-3,112 his3-11,15 ura3-1MAT* ***a*** *, isogenic to W303*	[32]
*esa1-414*	*ade2-1 trp1-1 can1-100 leu2-3,112 his3-11,15 ura3-1 esa1-1-414*::*URA3 MAT* **a**, *isogenic to W303*	*LPY4679* [27]
*esa1-L254P*	*ade2-1 trp1-1 can1-100 leu2-3,112 his3-11,15 ura3-1 esa1-L254P*::*URA3 MAT* **a**, *isogenic to W303*	*LPY5001* [27]
*BY4742*	*his3Δ1 leu2Δ0 met 15Δ0 ura3Δ0 MATa*	Euroscarf
*Δgcn5*	*gcn5::KanMX his3Δ1 leu2Δ0 met 15Δ0 ura3Δ0 MATa, isogenic to BY4742*	Euroscarf
*Δsas3*	*sas3::KanMX his3Δ1 leu2Δ0 met 15Δ0 ura3Δ0 MATa, isogenic to BY4742*	Euroscarf
*Δsas2*	*sas2::KanMX his3Δ1 leu2Δ0 met 15Δ0 ura3Δ0 MATa, isogenic to BY4742*	Euroscarf
*Δhat11*	*hat1::KanMX his3Δ1 leu2Δ0 met 15Δ0 ura3Δ0 MATa, isogenic to BY4742*	Euroscarf
*ΔRtt109*	*rtt109::KanMX his3Δ1 leu2Δ0 met 15Δ0 ura3Δ0 MATa, isogenic to BY4742*	Euroscarf
*ADA3-MYC*	*ADA3-MYC9::HIS3 ura3-1 leu2-3,112 his3-200 lys5 trp1 ada3::TRP1 MATa, * ***isogenic to W303***	[35]
*GCN5-MYC18*	*MATa GCN5-MYC::TRP1 ade2-1 can1-100 his3-11,15 leu2-3,112 his3-11, 15 ura3-1, * ***isogenic to W303***	[36]
*DF5gal4Δ*	*gal4Δ gal80Δ ura3-52 trp1-901 leu2-3,112 his3-200 MAT* ***a***	[53]

**Table 2:** High dosage of *GCN5* and *SPT8* partially suppress the *Ts^−^* phenotypes of replication factor mutants.

	High dosage gene	Growth at 23°C	Growth at 37°C
*orc2-1*	No episome	++++	−
	Control	++++	−
	GCN5	++++	++
	SPT8	++++	++
	SPT16	++++	−
	POB3	++++	−
*orc5-1*	No episome	++++	−
	Control	++++	−
	GCN5	++++	++
	SPT8	++++	++
	SPT16	++++	−
	POB3	++++	−
*mcm5-461*	No episome	++++	+/−
	Control	++++	+/−
	GCN5	++++	+++
	SPT8	++++	+++
	SPT16	++++	+/−
	POB3	++++	+/−

*orc2-1, orc5-1* and *mcm5-461* cell were transformed with high copy episomes (2 µm) carrying the shown genes and then grown at 23°C or 37°C. Growth was assessed by size of colony after 3 days at 23°C or after 5 days at 37°C.

### Plasmids

pGBKT7 (Clontech), pRS305 (SGD), pARS1wt and pARS1/-B23/G24 [20], pARS1wt/linker [21], pGAL4DBD-GCN5, pGAL4DBD-gcn5(KQL), pGAL4DBD-SAS2, pGAL4DBD-SAS3, pGAL4DBD-ESA1 and pGAL4DBD-HAT1 [22] have been described previously. pFW193 (*2 µm, URA3, SPT8*) is a gift from F. Winston. pTF125 (*2 µm, URA3, SPT16*) and pTF137 (*2 µm, URA3, POB3*) are gifts from T. Fromosa. pGBKT7-GCN5 was produced by replacing the large *HindIII* fragment containing the MCS with a PCR fragment containing *GCN5* under its own promoter.

Mini-chromosome stability assays with pARS1wt and pRS305 were performed as in [23], [24]. Suppression of plasmid loss in *W303, mcm5-461, orc2-1* and *orc5-1* cells was performed by double transformation with pARS1wt and pGBKT7 or pGBKT7-GCN5, respectively. Cells were selected on SC-trp-ura plates. Three individual colonies were inoculated in SC-trp to allow for retention of the pGBKT7 episomes and for loss of pARS1wt and grown for 20 generations. Each culture was serially diluted 1/10, and 5 ul of each dilution were spotted on both SC-trp-ura and on SC-trp plates. Assays with pARS1/-B23/GAL4 were performed by co-transforming in DF5gal4Δ cells with pARS1/-B23/GAL4 together with pGAL4DBD-GCN5, pGAL4DBD-GCN5(KQL), pGAL4DBD-SAS2, pGAL4DBD-SAS3, pGAL4DBD-ESA1 and pGAL4DBD-HAT1, respectively. Cells were selected on SC-his-ura plates, grown for 20 generations in SC-his and then plated on SC-his and SC-his-ura. In all cases plasmid loss was calculated as in [23].

### Chromatin Immuno-Precipitation (ChIP)

We used a protocol, which favors weaker protein interactions with chromatin. It includes a second cross-linking agent with a longer arm in the cross-linking step [25]. Briefly, *GCN5-MYC* or *ADA3-MYC* cells at OD_600_ = 1.0 in YPD were cross-linked with 1.6% formaldehyde for 30 minutes at 25°C and then suspended in 10 ml PBS/10 mM DMA(Pierce)/0.2%DMSO for another 30 minutes at 25°C. The cross-linking was quenched with 2.5 ml of 2.5 M glycine, cells were collected and crushed by glass beads in LB/IP buffer (50 mM Tris.HCl, 140 mM NaCl_2_, 5 mM EDTA, 1x PI cocktail (Sigma)) and sonicated by 12 bursts of 30 seconds at maximum output of Micronix cuphorn to produce fragments of about 500 bp. Chromatin was extracted in LP/IP plus 0.3% TX100 and 0.05% Na deoxycholate and immuno-precipitated overnight with anti-MYC beads (Sigma). The beads were washed three times with 1 ml LB/IP/0.5% TX100, 0.05% Na deoxycholate, one time with 1 ml LB/IP plus 350 mM NaCl, one time with 150 ul TE plus 10 mM NaCl (Final Wash) and eluted/uncrosslinked overnight in 150 ul TE/0.2% SDS at 65°C. After treatment with Proteinase K, DNA was extracted by Phenol/Chloroform and precipitated. The fragments of interest were quantified by PCR (after confirming linear range of amplification with crude genomic DNA) and ImageQuant software. Primer sequences are provided (Suppl. Table S1).

For the analysis of MCM loading on origins, *BY4247* and *Δgcn5* cells were grown at 23°C in YPD to OD_600_ = 1.0, arrested in Mitosis with 12 µg/ml Nocodazole for 3.5 hours, then released from mitotic arrest in fresh YPD. Aliquots of 100 ml were cross-linked and processed as above. Immunoprecipitation was conducted with a mixture of 2 ug each of anti-yMCM2 and anti-yMCM5 antibody (Santa Cruz) and Protein G beads (Sigma). Washing, elution, reversal of cross-linking and PCR were as described above.

### Restriction Nuclease Accessibility Assay

We used the protocol for targeted analysis of loci [26] with slight modifications. *BY4247* and *Δgcn5* cells (100 ml) were grown in YPD to OD_600_ = 1.0 and treated with Zymolase and pelleted. Spheroplasts were re-suspended in 5 pellet volumes of nuclease buffer (30 mM Tris.HCl pH 7.5, 10 mM MgCl_2_, 5 mM CaCl_2_, 50 mM NaCl, 1/100 volume of PI cocktail (Sigma)), split into 0.7 ml aliquots and incubated for 30 min at 37°C with 50 U of *BglII*, 50 U of *DraI*, 200 U of Micrococcal Nuclease (MNase) or no enzyme, respectively. The digestions were stopped with 1/5 volume of 5% SDS/100 mM EDTA. All samples were treated with Proteinase K, extracted with phenol/chloroform and precipitated. Subsequently, samples for the analysis of *ARS1* were digested overnight with 0.3 U per µg of DNA of each *EcoRI* and *EcoRV*. Samples for the analysis of *CEN4* were digested overnight with 0.5 U of *KspA1* and 0.2 U of *BglII* per µg of DNA. After ethanol precipitation, the DNA was resolved on 1.2% agarose gels and analyzed by Southern blot. The probe for *ARS1* was a PCR fragment adjacent to the *EcoRV* site. The probe for CEN4 was adjacent to the *BglII* site. The actual sequences of the probes are available upon request. Signals were acquired and quantified by Phosphorimager using ImageQuant software.

## Results

### Deletion of *GCN5* Impairs Mini-Chromosome Stability

**Figure 1:**
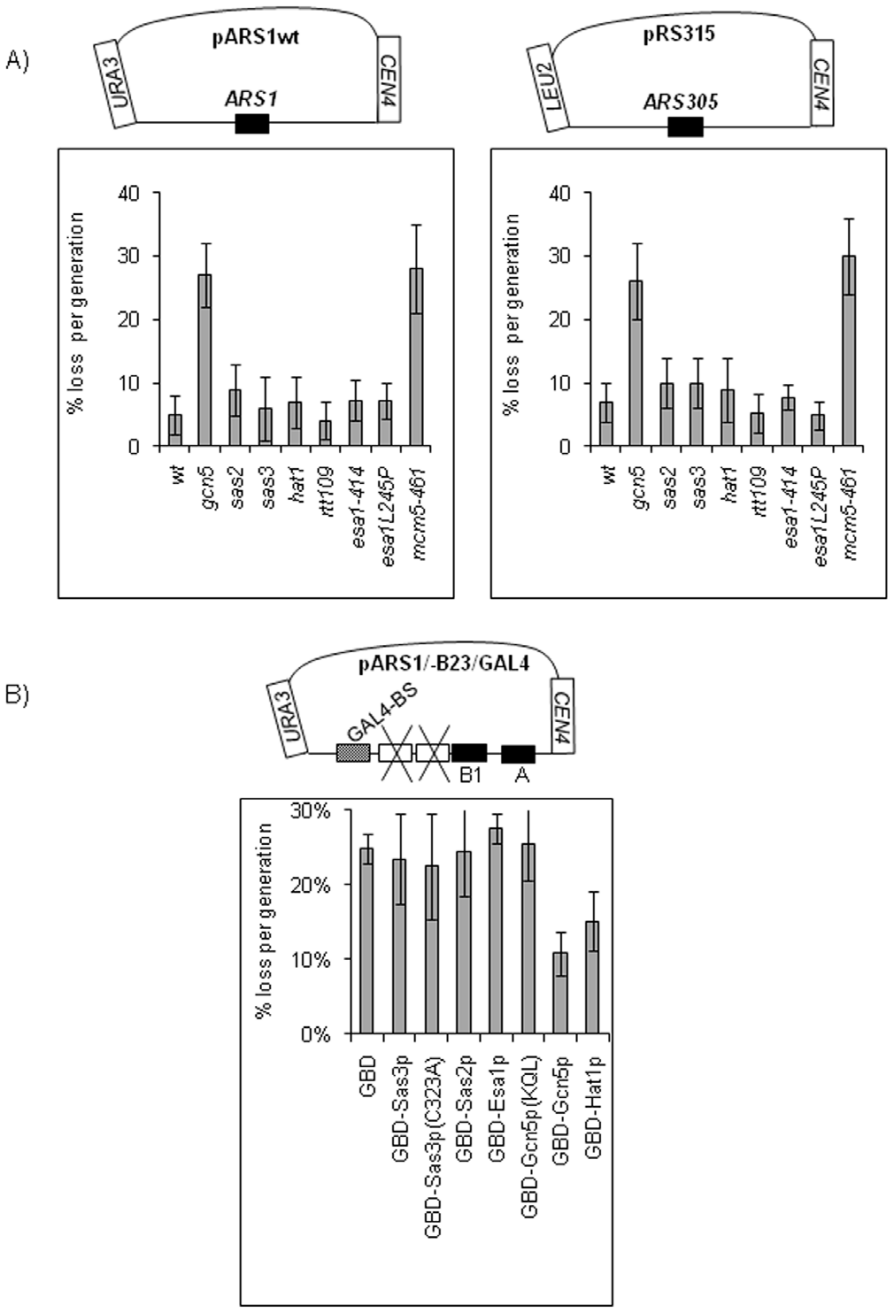
*GCN5* regulates mini-chromosome stability. **A. Disruption of** ***GCN5*** **increases the loss rate of** ***ARS1-*** **or** ***ARS305-*** **mini-chromosomes.** Maps of pARS1wt (*ARS1, CEN4, URA3*) and *pRS315 (ARS305*, *CEN4*, *LEU2*) mini-chromosomes are shown on the top of the graphs. The mini-chromosomes were transformed in the strains shown at the bottom of the graphs. After growth on selective plates (SC-ura or SC-leu, respectively) three colonies were transferred for 20 generations in rich medium (YPD) and plated on YPD and SC-ura or SC-leu, respectively. The proportions of cells with URA+ or LEU+ phenotypes were assessed and the average loss rate per generation was calculated (%) and plotted. Error bars represent standard deviations between the three parallel counts in the experiment. A typical outcome of one of three independent experiments is shown. **B. Tethering of Gcn5p increases the stability of pARS1/-B23/GAL4.** A diagram of pARS1/-B23/GAL4 *(ARS1-B2-B3+GAL4, CEN4, URA3)* is shown on the top. The B2 and B3 elements of *ARS1* are destroyed and a GAL4 binding site (GAL4-BS) is inserted next to B3. *DF5gal4Δ* cells were co-transformed with pARS1/-B23/GAL4 and episomes (*2 µm/HIS3*) expressing the GAL4 DNA binding domain fused to the proteins indicated below the graph. Gcn5p(KQL) lacks HAT activity. Average pARS1/-B23/GAL4 loss per generation and standard deviation were calculated as in Figure 1a. A typical outcome of one of two independent experiments is shown.

Mini-chromosomes are stably inherited (3–5% loss per generation) plasmids, which contain an *ARS*, a centromere (*CEN*) and a selection marker gene. Increased loss rate in non-selective medium is most frequently associated with flawed replication of the mini-chromosome independently of effects on other processes [24]. We used the mini-chromosome stability assay to test the involvement of several HATs in DNA replication. Strains with disrupted non-essential HAT genes (*Δgcn5*, *Δhat1, Δsas2, Δsas3* and *Δrtt109*) or mutations in the essential *ESA1* gene (*esa1-414*, *esa1L245P* [27]) were transformed with two different mini-chromosomes carrying *ARS1* (pARS1wt) or *ARS305* (pRS315). In Fig. 1A we show that the disruption of *GCN5* dramatically decreased the stability of both mini-chromosomes, while the deletion of the other HATs or the mutations in *esa1* had marginal effects. The level of mini-chromosome instability in *Δgcn5* cells was comparable to that of the *mcm5-461* strain, which harbors a mutation in one of the essential origin licensing factors, *MCM5* [28], [29].

### Tethering of Gcn5p Stimulates the Activity of an Impaired *ARS1*

The tethering of *Gcn5p* to a late origin (*ARS1412*) causes it to fire earlier [4]. We designed a similar tethering assay to directly compare the overall activity of several HATs on a mini-chromosomal origin. The mini-chromosome pARS1/-B23/G24 (Fig. 1B) carries *ARS1*, in which two of the auxiliary elements, *B2* and *B3*, are destroyed and a GAL4 binding site is inserted instead of *B3* [20]. These ablations render pARS1/-B23/G24 unstable to a point where its maintenance is critically dependent on the tethering of stimulators via the GAL4 binding site [20], [30]. To test the roles of different HATs on this crippled origin, we co-transformed DF*Δgal4* cells with pARS1/-B23/G24 plus a set of constructs expressing GAL4-fusions of Gcn5p, gcn5p(KQL) (a *GCN5* mutant lacking HAT activity), Sas2p, Sas3p, Esa1p and Hat1p, respectively, and measured the stability of pARS1/-B23/G24. In Fig. 1B we show that Gcn5p and Hat1p reduced the loss rate of pARS1/-B23/G24 from 25% to 10 and 12%, respectively. Since the theoretical maximum loss measurable by this assay is 50% for non-replicating plasmids and is in the range of 30% for very poorly replicating plasmids [24], these effects were considered significant. In contrast, Gcn5p(KQL), Sas2p, Sas3p and Esa1p did not reduce the loss rate of pARS1/-B23/G24 (Fig. 1B). We note that all GAL4-fusion constructs express active HAT proteins *in vivo* as demonstrated elsewhere [22].

### High Dosage of *GCN5* Suppresses Mini-Chromosome Instability in Replication Factor Mutants

**Figure 2:**
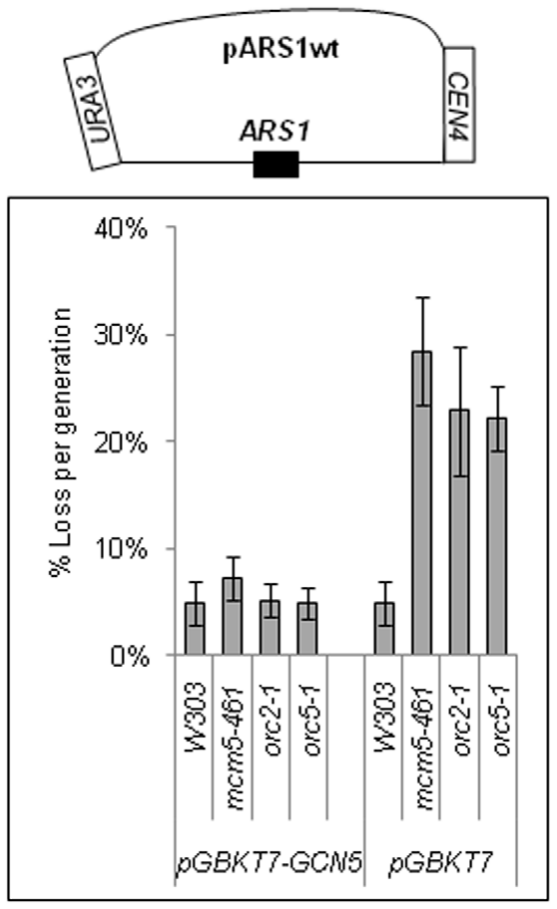
High copy number of *GCN5* suppresses mini-chromosome instability in *mcm5-461, orc2-1 and orc5-1*. *W303*, *mcm5-461, orc2-1 and orc5-1* cells carrying pARS1wt (*ARS1, CEN4, URA3*) were transformed with pGBKT7 (*2 µm/TRP1*) or pGBKT7-*GCN5*. After selection on SC-trp-ura plates three colonies were transferred for 20 generations in SC-trp and then plated on SC-trp and SC-trp-ura, respectively. The proportion of cells with URA+ phenotype was estimated and the average loss per generation of pARS1wt was calculated (%) as in Fig. 1A and plotted. A typical outcome of one of two independent experiments is shown.

In an earlier study we have shown that high dosage of *TRA1* (a component of the HAT complexes SAGA and NuA4) can suppress the *Ts^−^* phenotype of a strain with profound replication deficiency, *mcm5-461* [29]. We reasoned that if *GCN5* works in DNA replication, it may have a similar effect in this and other replication factor mutants. We supplemented the *mcm5-461* [29], *orc2-1* [31] and *orc5-1* [32] strains with stable high copy 2 µm episomes, which carry *GCN5* or a non-HAT component of the SAGA/ADA complex, *SPT8*. As controls, we used two genes, which encode components of the FACT complex, *SPT16* and *POB3*. FACT has been implicated in pol II transcription and in the elongation step of DNA replication, but not in origin activation [33], [34]. In Table 2 we show that high copy of *GCN5* and *SPT8* suppressed the conditional *Ts^−^* phenotypes of *mcm5-461, orc2-1 and orc5-1*. In contrast, *SPT16* and *POB3* had no effect. Next, we tested if the high dosage of *GCN5* in these mutants also suppressed the severe instability of mini-chromosomes. We co-transformed these strains with a test mini-chromosome (pARS1wt) and the *GCN5*-carrying episome and assessed the level of loss of pARS1wt. The high dosage of *GCN5* significantly improved the stability of pARS1wt relative to the control (Fig. 2). Hence, high dosage of *GCN5* specifically suppressed the replication deficiencies of *mcm5-461, orc2-1* and *orc5-1*.

### Gcn5p Associates with Origins of DNA Replication

**Figure 3:**
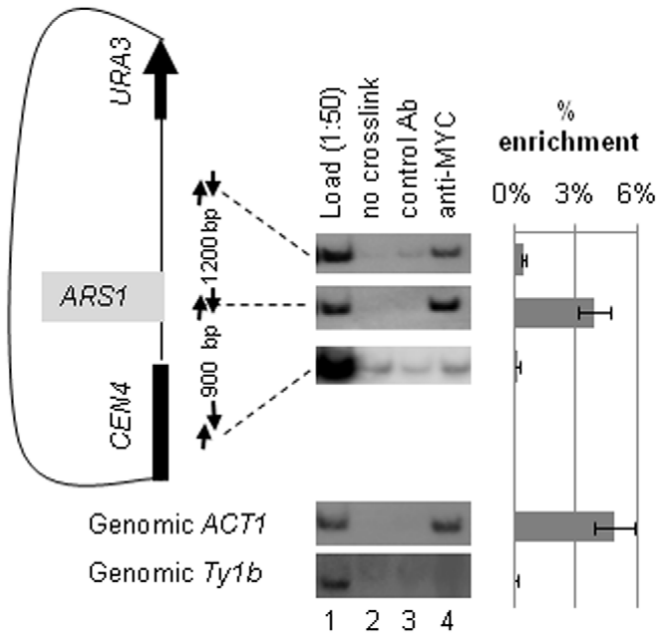
Gcn5p associates with mini-chromosomal *ARS1*. A map of the test mini-chromosome (pARS1wt/linker) is shown on the left. The distance between *ARS1* and *URA3* is 2.2 kb. The distance between the PCR fragments is shown between the pairs of arrows. *MYC-ADA3* cells harboring this mini-chromosome were cross-linked with formaldehyde and immunoprecipitated with anti-MYC antibodies. The precipitated DNA was quantified by PCR in triplicate. The per cent of signal from each fragment in the anti-MYC immuneprecipitate versus Load (diluted 1∶50) was calculated and plotted in the graph on the right together with the standard deviation in the three PCR reactions. Controls with genomic *ACT1* and Ty1b DNAs are shown below the mini-chromosome diagram. A typical outcome of one of two independent experiments is shown.

Next, we used ChIP assays to test if Gcn5p directly associates with origins of DNA replication. In the first set of experiments we used a modified pARS1wt/link mini-chromosome, which has been designed to minimize overlap of ChIP signals from *URA3* and *ARS1* ([21], see Fig. 3). It contains a 2.2 kb fragment of non-yeast DNA between *ARS1* and the *URA3* reporter. pARS1wt/link was introduced in the *ADA3-MYC* strain [35] and ChIP was performed with anti-MYC antibodies. Ada3p, Ada2p and Gcn5p build up a stable complex, which forms the catalytic core of all Gcn5p-containing HATs [35]. The amounts of precipitated mini-chromosomal *ARS1* only (one of the primers anneals to the plasmid backbone) and two flanking fragments that are positioned about 1 kb away from it were quantified by triplicate PCR. The data showed that about 4% of the mini-chromosomal *ARS1*, 0.6% of the fragment between *ARS1* and *URA3* and 0.1% of the fragment next to *CEN4* were immunoprecipitated by anti-MYC antibodies (Fig. 3, lane 4). In comparison, the anti-MYC antibodies precipitated none of the genomic *Ty1b* transposon (negative control) (Fig. 3, lower panel) and about 5% of the genomic *ACT1* (positive control). The non-crosslinked samples (Fig. 3, lane 2) and the samples without antibody (Fig. 3, lane 3) uniformly produced negligible signals with all loci tested. We concluded that Ada2p/Ada3p/Gcn5p specifically associates with the mini-chromosomal *ARS1*.

**Figure 4:**
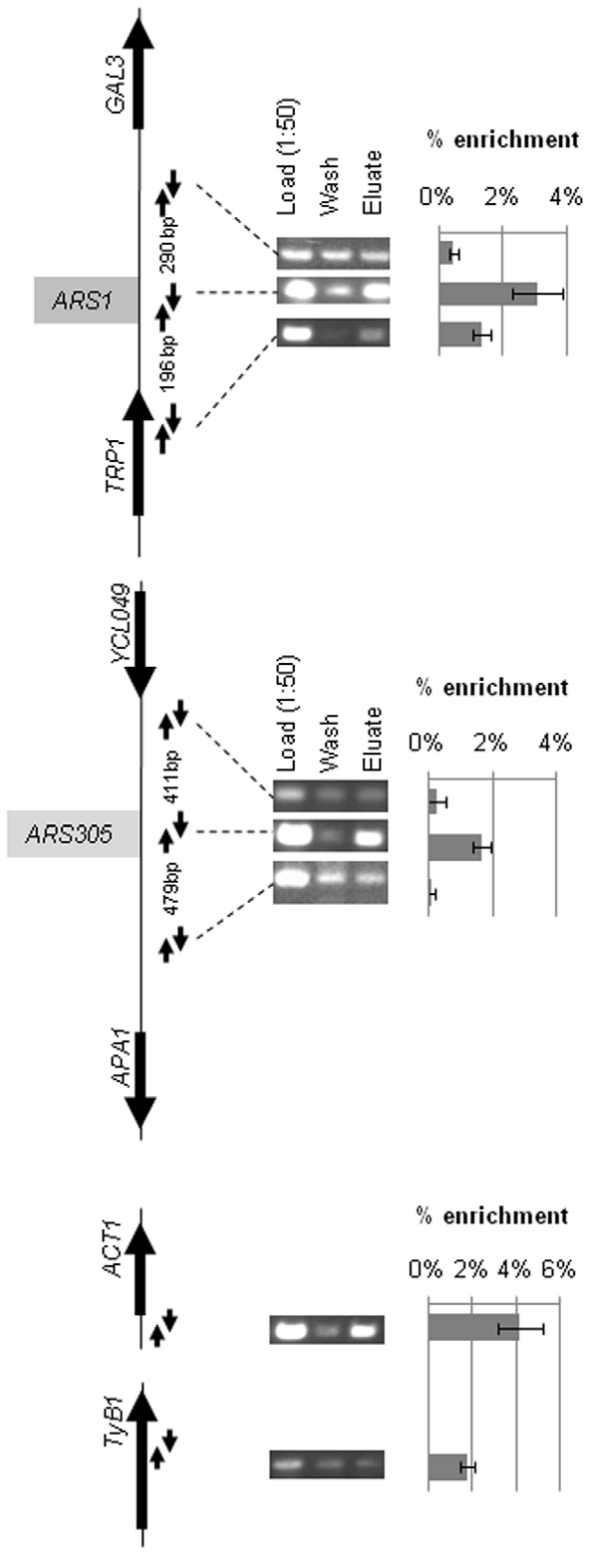
Gcn5p associates with chromosomal origins of DNA replication. Maps of the four genomic loci analyzed are shown on the left. Exponentially growing *MYC18-GCN5* cells were cross-linked with formaldehyde and immunoprecipitated with anti-MYC antibodies and the precipitated DNA was quantified by PCR in triplicate. The per cent of signal from each fragment in the anti-MYC immunoprecipitate versus Load (diluted 1∶50) was calculated and plotted in the graphs on the right together with the standard deviation in the three PCR reactions. The arrows in the diagrams represent the pairs of PCR primers used. The distance between the PCR fragments is shown between the pairs of arrows. A typical outcome of one of two independent experiments is shown.

In another set of experiments we analyzed the association of Gcn5p with chromosomal origins. We must note that the high gene density in the yeast genome does not provide the comfortable distances between genes and origins that were available on pARSwt/link. Because of this concern we focused on two extensively characterized origins with finely mapped *ACS* and auxiliary elements, *ARS1* and *ARS305*, in a *GCN5-MYC18* strain [36]. The *ACS* elements of these origins are located more than 500 base pairs (the average resolution of ChIP) away from the nearest promoter. In these assays we compared the signal from the core *ACS* to the signals from two flanking elements positioned between 200 and 500 bases away from the *ACS*s (see maps in Fig. 4). The relative amounts of each of the analyzed fragments in the Load, Wash and anti-MYC-Eluate fractions were quantified by PCR (triplicate). The per cent of signal in Eluate versus Load was calculated and plotted next the maps of *ARS1* and *ARS305* (Fig. 4). In addition, we tested association of Gcn5-MYCp to two control loci: the *ACT1* gene and the repressed *Tyb1* transposon (Fig. 4).

The ChIP experiments consistently produced higher signals from the *ACS* elements of *ARS1* and *ARS305* as compared to the flanking fragments. The moderately high signal of 1.3% at the *ARS1* flanking element towards *TRP1* probably reflects its proximity to the origin (196 bp) or strong association of Gcn5p to the upstream *TRP1* gene. The recovery of DNAs in the immunoprecipitates represented 2.9% for *ARS1* and 1.8% for *ARS305* of the total DNA analyzed, while the recovery of *ACT1* (positive control) DNA was 4.3% (Fig. 4). As expected, no association of Gcn5p to the repressed *Ty1b* element was observed (Fig. 4). These weaker but still noticeable chromatin immunioprecipitation signals from origins relative to promoters are in tune with the observations in [36]. In this comprehensive genome-wide mapping of Gcn5p association (data available on http://frodo.wi.mit.edu/cgi-bin/yeast_histones/gbrowse.cgi/yeast_histones) moderately higher signals of Gcn5p binding at *ARS1* and *ARS305* (and also at *ARS501, ARS606, ARS1021, ARS1414, ARS1618*) relative to the immediate neighboring regions can be observed. However, these peaks were below the preset threshold of the computational analysis and were not discussed in [36].We should also mention that many *ARS* reside within short distance from gene promoters thus adding to the problematic interpretation of such weak signals. We completely agree that the genome-wide approach in [36] has not identified association of Gcn5p to *ARSs*. Nevertheless, their data are in tune with our focused approach and support the notion of weak association of Gcn5p to origins. We also need to note that we used a protocol with two cross-linkers (formaldehyde and DMA, [25]) that enhances weak ChIP signals as compared to a single cross-linker (formaldehyde) in [36]. In summary, the results in Figure 4 showed specific, but weaker association of Gcn5p to chromosomal origins relative to the better isolated mini-chromosomal *ARS1* origin (Fig. 3). This outcome could be a consequence of the expected higher background signal on chromosomal origins. The alternatively explanation is that for some unknown reason the association of Gcn5p to the mini-chromosomal *ARS1* origin is intrinsically stronger.

### Deletion of *GCN5* Perturbs Chromatin Structure of a Chromosomal Origin

We reasoned that if the weak association of Gcn5p to chromosomal origins is functionally relevant, the deletion of *GCN5* would affect their chromatin structure. Initial analyses by limited MNase digestion showed the existence of hypersensitivity sites in the vicinity of *ARS1*, *ARS305* and *ARS501* (not shown). However, the differences between *Δgcn5* and *wt* cells were hard to assess because this enzyme also cleaves in the broader area of the analyzed loci. It is noteworthy that a similar DNAase accessibility assay has failed to detect variations in the structure of *ARS1* between *wt* and *orc2-1* cells [18], yet it is known that *ARS1* activity is severely impaired in *orc2-1* cells [31]. We concluded that the MNase or DNAase sensitivity assays are inadequate for analysis of *ARS*. Therefore, we tailored a focused restriction enzyme (*BglII*) accessibility assay [26] for the analysis of *ARS1* on chromosome IV. *BglII* cleaves *ARS1* between the B2 and B3 elements [20]. Briefly, equal amount of spheroplasts from exponentially growing *wt* or *Δgcn5* cells, respectively, were lysed and exposed to excess of *BglII*. Under these conditions the cleavage of sites depends on site accessibility thus representing the level of chromatin compactness of the tested locus. Control samples were incubated without *BglII* to monitor for endogenous nuclease activity. In addition, to distinguish specific signals during the indirect end-labeling, excess of MNase was added in parallel samples of lysed spheroplasts. Subsequently, total DNA from all samples was purified and a second complete digestion was performed with *EcoRI* and *EcoRV*, which cut on either side of *ARS1*. Finally, the produced DNA fragments were analyzed by indirect end-labeling with a probe, which anneals 475 bp away from the *BglII* site. The ratio of un-cleaved *EcoRI-EcoRV* versus *BglII*-cleaved fragment was used as a measure for the accessibility to *ARS1* and therefore for chromatin compactness of the analyzed locus.

**Figure 5:**
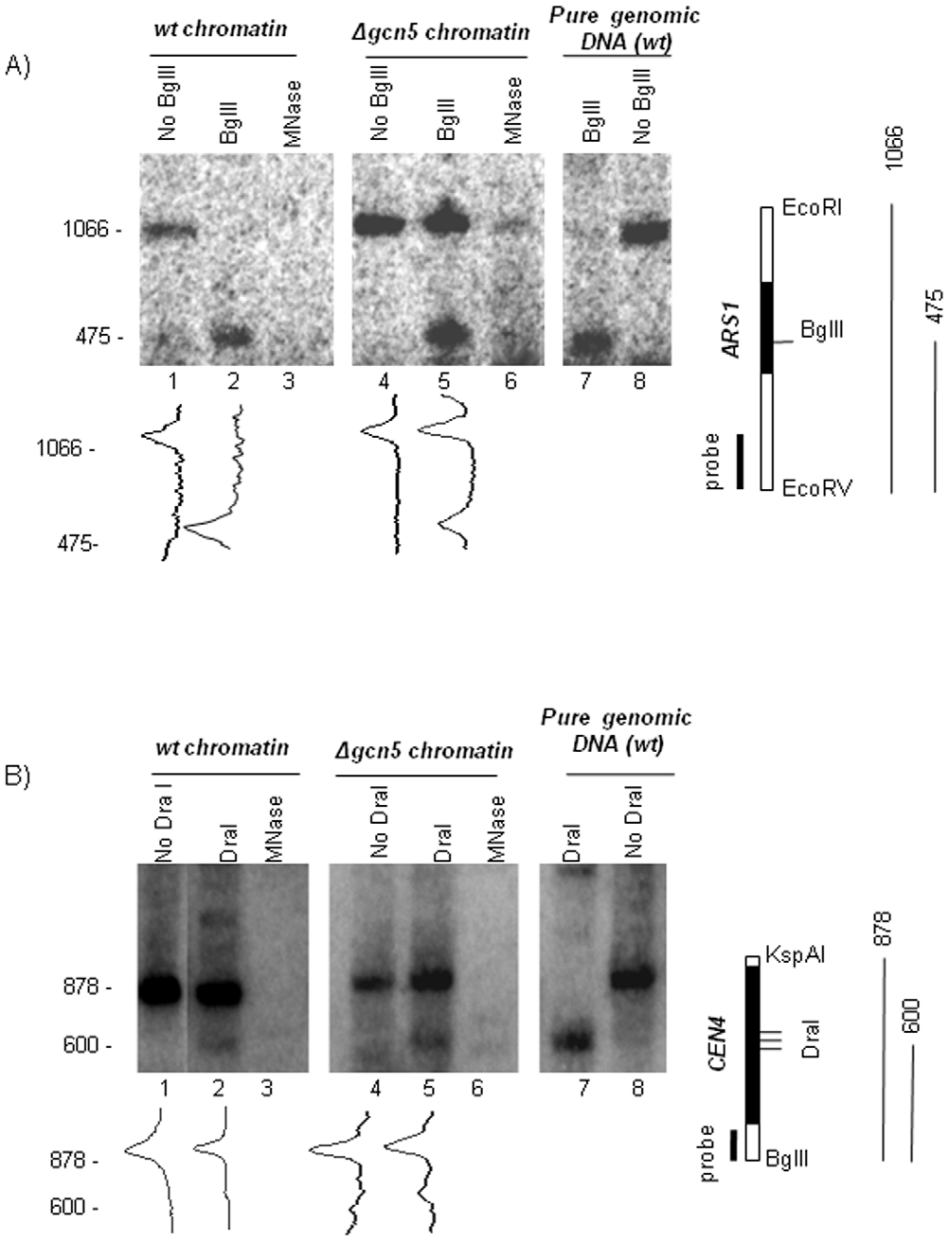
Chromatin structure of *ARS1* and *CEN4* in *Δgcn5* cells. Spheroplasts from *Δgcn5* and the isogenic wild type cells (*BY4741*) were lysed and exposed to excess of *BglII*, *DraI* or *MNase* as indicated on top of each lane. After purification and complete digestion with restriction enzymes that cut at each side of the analyzed loci (shown on the right), DNA fragments were analyzed by Southern blot with the probes shown on the right. A typical outcome of one of three independent experiments is shown. **A. Analysis of** ***ARS1*** **.** Diagram of the *ARS1* locus on chromosome IV with the positions of *ARS1*, of the probe for Southern blot and of the *BglII*, *EcoRV* and *EcoRI* sites is shown on the right. The *BglII*-cleaved (475 bp) and uncleaved (1066 bp) fragments are also shown. Density graphs of lanes 1, 2, 4 and 5 are shown underneath. **B. Analysis of** ***CEN4*** **.** Diagram of the *CEN4* locus on chromosome IV with the positions of *CEN4*, of the probe for Southern blot, of the *BglII* and *KspAI* sites and the three juxtaposed *DraI* sites is shown on the right. The *DraI*-cleaved (600 bp) and uncleaved (878 bp) fragments are also shown. Density graphs of lanes 1, 2, 4 and 5 are shown underneath.

Using this assay we reproducibly observed high accessibility to the *BglII* site in *ARS1* in *wt* cells (Fig. 5A, lane 2), while the parallel samples from *Δgcn5* cells (Fig. 5A, lane 5) consistently showed moderate accessibility. The bands corresponding the *BglII*-cleaved (475 bp) and un-cleaved (1066 bp) fragment were abolished by treatment with MNase. These results clearly show that in *Δgcn5* cells *ARS1* acquires a compact structure resistant to nuclease digestion.

### Deletion of *GCN5* Has a Relatively Minor Effect on the Structure of *CEN4*

An earlier study has implicated *GCN5* in the regulation of centromeres [37]. More specifically, the study has shown increased accessibility of *DraI* sites in centromeres in *Δgcn5* cells. We attempted to evaluate the extent of chromatin modifications at origins and centromeres in *Δgcn5* cells using a similar *DraI*-accessibility assay for the analysis of *CEN4*. Samples from *wt* and *Δgcn5* cells prepared in parallel with the samples for Fig. 5A were digested with *DraI* and processed accordingly with *KspI* and *BglII*. Next, indirect end-labeling with a probe annealing 600 bp from the three juxtaposed *DraI* sites in *CEN4* was performed (Fig. 5B). In these experiments we consistently observed fairly similar levels of *DraI* cleavage in both *wt* and *Δgcn5* cells (Fig. 5B, lanes 2 and 5). Calculation of the intensity of the cleaved (600 bp) and uncleaved (878 bp) *CEN4* fragments in Fig. 5B showed that 14% and 18% of the *DraI* sites were digested in *wt* cells or *Δgcn5* cells, respectively. In agreement, the accessibility to *DraI* in [37] was similarly estimated at 9% in *wt* cells and 13% in *Δgcn5* cells.

In summary, our assays have demonstrated that the deletion of *GCN5* produces minor alterations in the chromatin structure of *CEN4* and major alterations of *ARS1*.

### Deletion of *GCN5* Impairs the Assembly of Pre-Replicative Complexes on Origins in G1

Earlier studies have shown that the lack of *GCN5* delays progression through G_2_ and Mitosis after synchronization with α-factor [37], [38]. When cells are synchronized in Mitosis, they also progress slower through G1/early S phase and display later appearance of buds as compared to wild type cells (Figure S1). We considered that an event in Mitosis or G1 that is important for regulation of origins could be impaired in *Δgcn5* cells.

**Figure 6:**
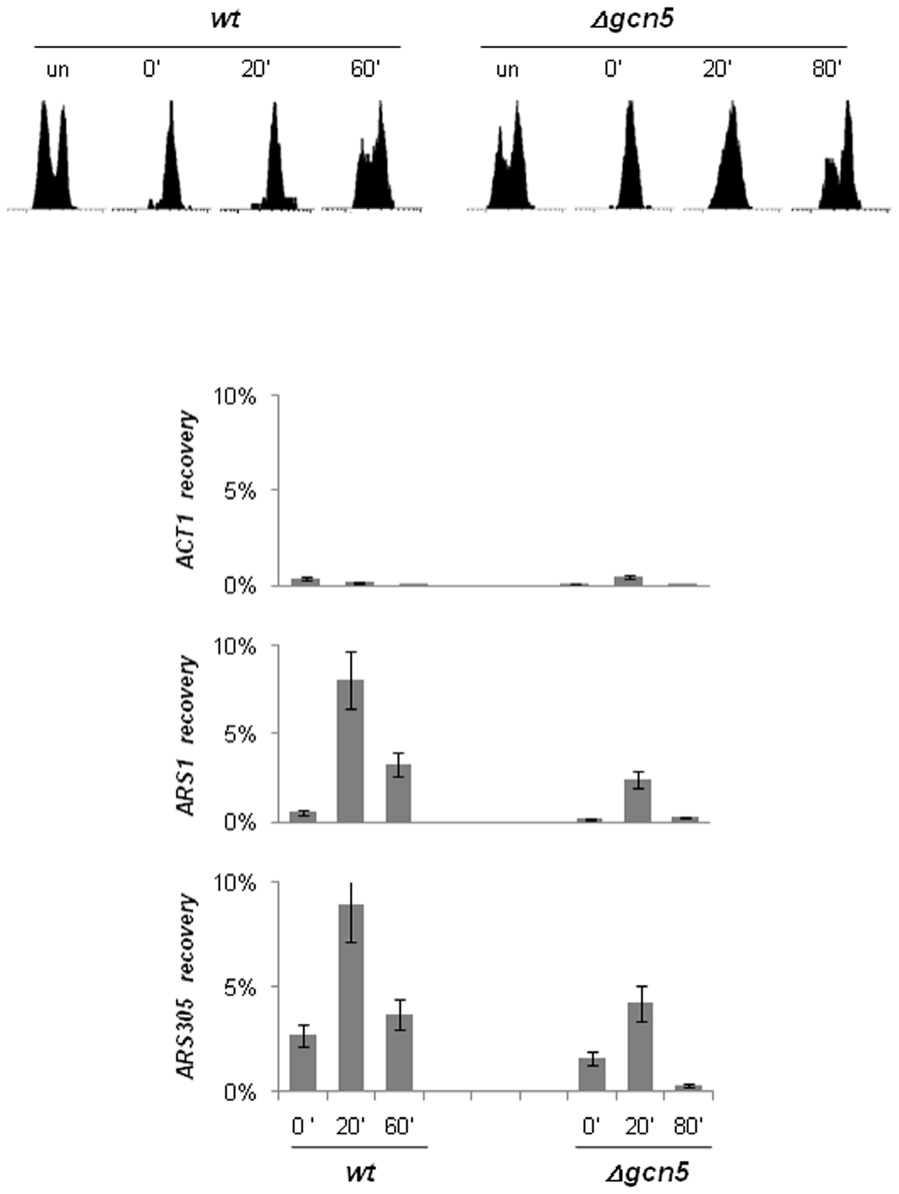
Reduced association of MCM proteins to *ARS1* and *ARS305*. Wild type *(BY4742)* and *Δgcn5* cells were arrested in Mitosis by12 µg/ml Nocodazole for 3.5 hours and released in YPD. Wild type cells were harvested at 0, 20 and 60 min past Nocodazole release. *Δgcn5* cells were harvested at 0, 20 and 60 min past Nocodazole release. Progression through the cell cycle as monitored by Propidium Iodide staining/FACS is shown on the top. Crosslinked chromatin was isolated and immunoprecipitated with anti-MCM2 and anti-MCM5 antibodies. The *ACS* fragments of *ARS1* and *ARS305* and a fragment from *ACT1* were amplified by PCR (triplicate) and quantified. The per cent of signal from each fragment in the anti-MYC immunoprecipitate versus Load was calculated and plotted. A typical outcome of one of two independent experiments is shown.

Shortly after exit from Mitosis the origins assemble and “license” pre-replicative complexes. This “licensing” is highlighted by the loading of the MCM proteins [1]. Consequently, the binding of MCM proteins to origins is used as an assessment for the efficiency of assembly of pre-replicative complexes [39]. We tested the hypothesis that the negative effect on DNA replication in *Δgcn5* cells is linked to impaired loading of MCM proteins. We synchronized *wt* and *Δgcn5* cells in Mitosis by Nocodazole and then released them in YPD. Cells were cross-linked at 20 min past release when both cell strains are in G1 and show no buds (Fig. 6 and Suppl. Fig. S1). Another sample was collected and cross-linked 60 or 80 min past release for *wild type* or *Δgcn5* cells, respectively. Cross-linked chromatin was sheared, extracted and immunoprecipitated with a mixture of anti-MCM2 and anti-MCM5 antibodies (Santa Cruz). We have found that this mixture of anti-peptide antibodies is more efficient in recovery of origin DNA as compared to the individual antibodies used alone [40], [41]. Finally, the immunoprecipitated DNA was analyzed by quantitative PCR with primers for *ARS1* and *ARS305*. Consistent with many previous reports (reviewed in [1]), in *wt* cells we saw significant enrichment of anti-MCM immunoprecipitated *ARS1* and *ARS305* DNA at the 20^th^ min past release (G1) with significant decrease of the signals at the 60^th^ min (S) (Fig. 6). In comparison, *ACT1* showed very little association with MCM proteins at the time points of our experiment. A similar pattern of MCM binding to origins was observed in *Δgcn5* cells except that the S phase sample was collected at the 80^th^ minute to compensate for the slower progression through the cell cycle. Importantly, in *Δgcn5* cells the recovery of *ARS1* and *ARS305* DNA in G1 was two-three fold lower as compared to *wt* cells (Fig. 6). These results demonstrate significant deficiency in the assembly of pre-replicative complexes in *Δgcn5* cells.

## Discussion

*In S.cerevisiae*, ten HATs have been described to date [42], [43], [44]. Several genome-wide studies on gene expression [45], [46], [47], [48] and chromatin association [36], [49] have provided significant details on the engagements of the HAT complexes SAGA, TFIID, Mediator and NuA4 at gene promoters. However, these papers have not suggested the participation of any of these HATs in DNA replication. On the other hand, focused studies of origins or studies on protein interactions have tentatively linked *HAT1* [19] and *SAS2* [18] to DNA replication. In the present study we have compared the effects on DNA replication of deletions of *HAT1, SAS2, SAS3, GCN5* and *RTT109* and mutations in the essential *ESA1* gene. These assays clearly outlined a major replication deficiency in the *Δgcn5* strain with minor effects in the others (Figures 1 and 2). All subsequent analyses supported the notion that *GCN5* is a positive regulator of origins. We postulate that *GCN5* counteracts the negative effects of HDACs such as *SIR2* and *RPD3* [4], [5], [6]. Our observations expand the functional repertoire of *GCN5*, which has already been implicated in gene expression [43] and also in centromere function [37].

*GCN5* has been extensively characterized as a global regulator of gene expression [36], [47], [50], [51]. Is it then possible that all effects we have observed are indirect via deregulated gene expression? Several arguments reduce the likelihood of this possibility. Firstly, we have detected credible signals of direct association of Gcn5p with a mini-chromosomal *ARS1* (Fig. 3). Whereas the chromosomal *ARS1* and *ARS305* do not provide the luxury arrangement and distances between the genes and origins on the mini-chromosome, signals from direct Gcn5p association at these loci have been detected by us (Fig. 4) and others [36]. Finally, transcriptome analysis in *Δgcn5* cells has revealed a bias towards the regulation of stress induced genes, but not towards genes responsible for DNA replication factors [47]. We therefore favor the idea that *GCN5* directly regulates DNA replication.

The fact that Gcn5p weakly and possibly only transiently associates with origins (Fig. 4) suggests that its effects on origins are produced by global activity rather than targeted recruitment as is the case on promoters [36]. This idea is in tune with the notion that the negative origin regulators *SIR2* and *RPD3* are likely to act by global deacetylation [4], [5], [6]. A recent study suggests that at least in the case of *RPD3* some targeted recruitment to origins is possible, but the mechanism remains unknown [7]. Regardless of whether the mode of action of Gcn5p is targeted or global, the effect of *GCN5* on the chromatin structure of origins is substantial as shown in Fig. 5A. Another point of consideration regarding the mechanism of action of *GCN5* is the observed deficiency of assembly of pre-replicative complexes in *Δgcn5* cells (Fig. 6). This result suggests that *GCN5* could have a major role in chromatin de-condensation, which in turn affects the licensing of origins. Studies in human cells have allocated a similar role in G1 for HBO1 [10], [11], [12]. At this point it is not clear if in human cells HBO1 is the only HAT engaged in G1 or if *GCN5* also participates. As for *S.cerevisiae*, the remote functional homologue of HBO1, *SAS2*, shows little activity consistent with a major role in replication (Fig. 1). On the other hand, the deletion of *HAT1* seems to have only minor effect on mini-chromosome stability (Fig. 1A), but strongly stimulates an origin when tethered to it (Fig. 1B). This observation and the data in [19] indicate that *HAT1* could be involved in replication but the assays employed by us did not properly reveal such function. Finally, the proposed role of *GCN5* in chromatin de-condensation and facilitation of pre-replicative complexes in G1 does not at all exclude additional roles in S phase as activator of origins or during elongation.

A very recent study has shown that in human cells *GCN5* acetylates the key component of pre-replicative complexes CDC6 thus modulating its subsequent phosphorylation by Cyclin A-CDKs [52]. It is not clear if similar acetylation of Cdc6p by Gcn5p takes place in *S. cerevisiae*. It is tempting to speculate that at least some of the effects we observe here are consequence of not only histone acetylation, but of acetylation of Cdc6p as well. This question remains open until clear link between *GCN5* and *CDC6* is established.

Our data suggests that *GCN5* has a major role in DNA replication. Nevertheless, *GCN5* is not an essential gene so it is obviously not the only HAT involved. In support, the deletion of *sas2*, *sas3* and *hat1* and the *esa1* mutants displayed slightly higher mini-chromosome instability as compared to wild type cells, but significantly lower as compared to *gcn5* (Fig. 1A). It is conceivable that these HATs also play roles in the same steps, which are regulated by *GCN5*. In particular, it is not clear to what extent the mutations in *ESA1* impair its function thus rendering its role in DNA replication far for from being excluded. It is noteworthy that *ESA1* and *GCN5* show a significant level of functional overlap on gene promoters [36].

Another issue stemming from our study is the possible role of *GCN5* on centromeres [37]. As mentioned earlier, GCN5 has been implicated as a regulator of chromatin structure and in the function of centromeres [37]. Indeed, double deficiency of the *ARS* and *CEN* elements can explain the magnitude of *Δgcn5* effect in Fig. 1. Whereas such scenario is not unlikely, our data on two chromosomal origins and a centromere shows that the alteration in chromatin structure is significant at origins and moderate to low on centromeres (Fig. 5). Even more, *GCN5* strongly suppresses the replication deficiency phenotype of the *mcm5-461, orc2-1* and *orc5-1*strains (Fig. 2). Hence, we suspect that *GCN5* is a major positive replication factor independently of its activity on centromeres.

In summary, we have assigned a new role for *GCN5*. We have also introduced a new HAT in the regulation of DNA replication. Future studies should focus on the interplay between *GCN5* and other HATs in modulating the activity of origins and the subsequent steps in DNA replication.

## Supporting Information

Figure S1 (0.06 MB TIF)

Table S1 (0.03 MB DOC)
