# Supplementary figures and images for: GCN5 Is a Positive Regulator of Origins of DNA Replication in Saccharomyces cerevisiae
Source: PLoS One. 2010 Jan 29;5(1):e8964. doi: 10.1371/journal.pone.0008964 (PMC2813283; doi:10.1371/journal.pone.0008964)

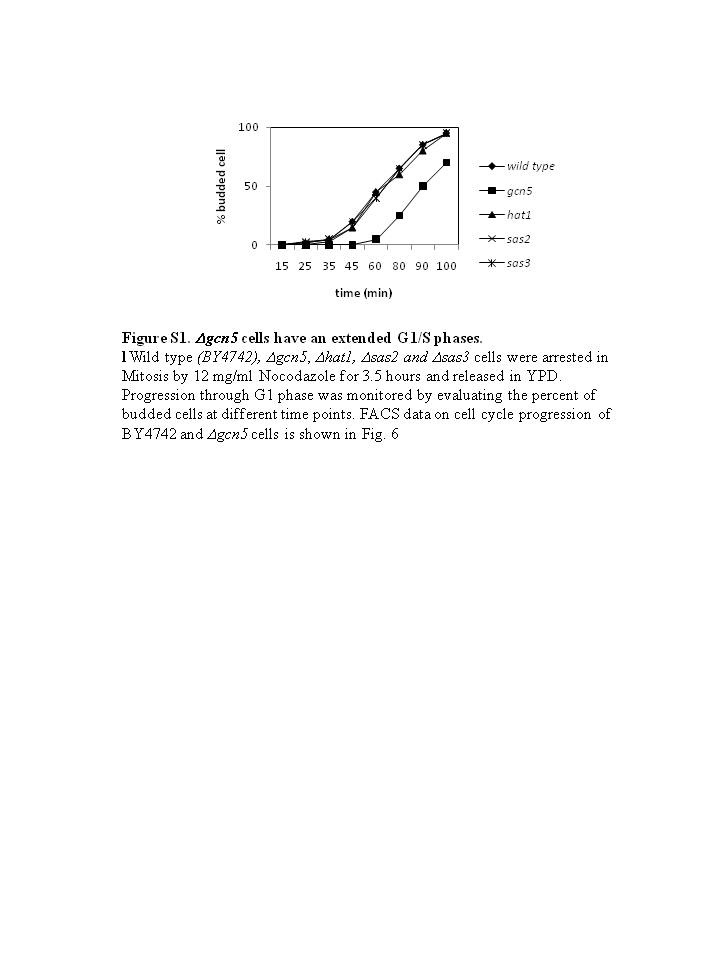

Supplement: Figure S1 — (0.06 MB TIF) [file pone.0008964.s001.tif]
