# Supplementary material for: GCN5 Is a Positive Regulator of Origins of DNA Replication in Saccharomyces cerevisiae
Source: PLoS One. 2010 Jan 29;5(1):e8964. doi: 10.1371/journal.pone.0008964 (PMC2813283; doi:10.1371/journal.pone.0008964)
Supplement: Table S1 — (0.03 MB DOC) [file pone.0008964.s002.doc]

Table S1. Sequences of the primers used:

| **Name** | **Oligonucleotide** (5’→3’) | **Coordinates of amplified fragment** |
| --- | --- | --- |
| *ARS1 – TRP1* forward  *ARS1 – TRP1* reverse | GATATCGTCCAACTGCATGGAGA  TCCCACCTGCTTCTGAATCA | Chr IV: 462120 - 462290 |
| *ARS1-ACS* forward  *ARS1-ACS* reverse | GTGGAGACAAATGGTGTAAAAGAC  GCGGTGAAATGGTAAAAGTC | Chr IV: 462446 - 462710 |
| *ARS1 – GAL1* forward  *ARS1 – GAL1* reverse | CATAAACACCATCAGCCTCAAG  TGAAGGAGCATGTTCGGCACA | Chr IV: 463003 - 463173 |
| *ARS305* forward  *ARS305* reverse | GCTTCTTGCTGTAGGTTATGGGA  TCGTCCTGTCAAAAGGCGAA | Chr III: 39100 - 39277 |
| *ARS305 –ACS* forward  *ARS305 – ACS* reverse | AGTTTCATGTACTGTCCGGTGTGAT  CGTTTTTAGCCCCCGTGTAAG | Chr III: 39449 - 39647 |
| *ARS305 - YCL049* forward  *ARS305 - YCL049* reverse | CTGGATAAACACCGCTACAAT  TCTCGGTGCCATATAATTGG | Chr III: 40058 - 40319 |
| *ACT1 forward*  *ACT1 reverse* | GATGAAGCTCAATCCAAGAGAGG AGTTGGTGGAGAAAGAGTAACCACG | Chr IV: 53736 - 54215 |
| *Ty1 forward*  *Ty1 reverse* | ACCACAGCGGATTCCCGAGCACATGGAG  TTGATCTAATCTGGATTCCTTGCCCTG | Multiple Ty loci |
